# Functional stratification and enzymatic arrangement in microbial communities across a hypersaline depth gradient

**DOI:** 10.3389/fmicb.2025.1624058

**Published:** 2025-09-17

**Authors:** Claudia Hoepfner, Daniel Guzmán, Boris Vidal-Veuthey, Valeria Foronda, Antonia Beggs, Juan P. Cárdenas, Virginia A. Vargas, Fernando D. Alfaro

**Affiliations:** ^1^Faculty of Sciences and Technology, Center of Biotechnology, Universidad Mayor de San Simón, Cochabamba, Bolivia; ^2^Faculty of Interdisciplinary Studies, GEMA Center for Genomics, Ecology and Environment, Universidad Mayor, Huechuraba, Chile; ^3^Faculty of Science, Engineering and Technology, Center for Genomics and Bioinformatics, Universidad Mayor, Santiago, Chile

**Keywords:** extreme environments, Andean Cold Deserts, extremozymes, shotgun metagenomics, extremophile biotechnology, bioprospecting

## Abstract

Extreme environments comprise a significant portion of Earth’s terrestrial surface, posing challenges, such as extreme temperatures, pressure, pH extremes, oxygen and nutrient scarcity, and high salinity. Hypersaline ecosystems, such as those in the Andean Cold Deserts, exemplify extreme environments where microbial life has evolved specialized survival mechanisms. The Central Andean Mountains host extensive salt flats exposed to extreme temperature fluctuations, intense ultraviolet radiation, and high soil salinity. While most studies focus on surface layers, the impact of soil depth on functional diversity remains poorly understood. This study utilized shotgun metagenomics and functional annotation to explore enzymatic diversity across a 8-meter depth gradient in the Uyuni Salt Flat aiming to understand microbial adaptations to depth and abiotic stress. Our findings revealed a complex, stratified microbial ecosystem. Surface layers showed high abundance of amylases, enzymes that degrade accessible carbohydrates, likely derived from photosynthetic communities or surface-imported organic matter. These patterns suggest a dominance of strategies for rapid carbon decomposition. Intermediate depths exhibited elevated lipase and peroxidase activity, reflecting the presence of complex lipids and oxidative stress management, essential for survival in oxygen-limited, high-salinity zones. Lipase support lipid utilization as a carbon source, while peroxidase activity points to redox adaptations for microbial resilience under fluctuating oxidative conditions. Deeper sediment layers showed a shift toward protease and peptidase activity, indicating organic nitrogen recycling in nutrient-deprived environments and suggesting an efficient protein degradation system among halophilic archaea. Peroxidases remained abundant even at these depths, supporting sustained redox regulation and biogeochemical cycling thus enabling microbes to manage redox imbalances in high-salinity, low-oxygen settings. The enzymatic diversity across the depth gradient demonstrates functional stratification and remarkable microbial adaptability to hypersaline conditions. This functional resilience underpins nutrient cycling and organic matter decomposition deep in the salt flats. Notably, the identified halophilic enzymes, stable and active under high-salinity conditions, hold significant potential for biotechnological applications. This study contributes to our understanding of microbial life’s complexity in hypersaline environments, enhancing our ability to harness extremophilic enzymes for biotechnological applications while underscoring the ecological value of these unique habitats.

## 1 Introduction

Drylands cover over 45% of the Earth’s terrestrial surface (Prăvălie, 2016). Salt flats represent a small yet distinct fraction of global drylands, typically found in arid and semi-arid regions (Svendsen, 2003). These landscapes often originate as remnants of ancient lakes that dried up over millennia through climatic, hydrological, and geomorphological changes, formed from water accumulation in closed basins with little drainage. These ecosystems are generally characterized by periodic inundation and evaporation due to the high evaporation rates in arid climates, resulting in sediment deposition and the progressive accumulation of salt (McKnight et al., 2023).

Such is the case of the Uyuni Salt Flat, located in the southwest of the Bolivian Altiplano (coordinates 20°08′01.59″S 67°29′20.88″W). It is the largest salt flat on Earth (Svendsen, 2003), and a unique hypersaline system shaped by extreme environmental pressures, including temperature and humidity fluctuations, intense UV radiation, and elevated concentrations of chaotropic agents such as MgCl_2_, LiCl, and NaBr (Risacher and Fritz, 1991; Pecher et al., 2020). These conditions select highly specialized extremophiles capable of thriving under simultaneous stressors, including high salinity, desiccation, nutrient scarcity, and strong oxidative pressures (Rothschild and Mancinelli, 2001). Despite such harshness, all three domains of life are present, with bacteria and archaea being the most dominant and functionally relevant taxa (Shu and Huang, 2022).

Microbial communities in hypersaline environments display remarkable resilience, often through unique metabolic strategies and enzymatic adaptations that enable them to survive under extreme ionic and osmotic stress (Madern et al., 2000; Gunde-Cimerman et al., 2018). These adaptations often include the production of halophilic enzymes that maintain functionality and stability in high-salt concentrations, facilitating metabolic processes essential for survival (Madern et al., 2000; Broman et al., 2022; DasSarma and DasSarma, 2015; Oren, 2024; Mevarech et al., 2000). Furthermore, these communities exhibit enhanced stress response mechanisms, such as DNA repair and oxidative stress tolerance (Martínez et al., 2021; Thompson et al., 2022; Demergasso et al., 2004; Martínez et al., 2022). These features are crucial for survival and contribute significantly to biogeochemical cycling and potential biotechnological applications (McGenity et al., 2000).

Soils and saline sediments host a great abundance of microorganisms, representing a significant portion of Earth’s total living biomass (Banerjee and van der Heijden, 2023). These microbiomes are recognized as some of the most taxonomically rich and functionally complex on the planet (Fierer, 2017) often containing thousands of microbial species and sustaining numerous ecosystem services that influence a broader environmental health (Bardgett and van der Putten, 2014). Central to these ecosystem functions is the diverse enzymatic toolkit that microbes deploy to drive the turnover of organic carbon and nitrogen. Complex carbon compounds are enzymatically broken down by specialized hydrolytic enzymes. Likewise, the acquisition of nitrogen relies on microbial enzymes capable of processing both peptide and non-peptide nitrogen sources (Farzadfar et al., 2021). The enzymatic breakdown of these complex carbon and nitrogen compounds into simpler forms represents a fundamental, often rate-limiting step in organic matter turnover and nutrient availability in extreme environments (Uwituze et al., 2022; Zhao et al., 2023).

Our understanding of soil microbiome relies on studies conducted in surface layers (Naylor et al., 2022). However, subsurface layers have distinct biological features influenced by depth-dependent physicochemical properties, such as nutrient levels, physical structure, and edaphic factors, which vary with depth (Shu and Huang, 2022). Environmental parameters vary considerably with increasing depth and giving rise to functionally specialized assemblages. Depth-related environmental gradients strongly influence the enzymatic repertoire of microbial communities (Broman et al., 2022; Bryanskaya et al., 2022; Naylor et al., 2022; Rchiad et al., 2022; Dove et al., 2021). Halophilic archaea and bacteria may express extremozymes—such as proteases, lipases, and dehydrogenases- optimized for activity and stability under high ionic strength. These enzymes often feature acidic surface residues and compact conformations, enhancing resistance to denaturation. Functional stratification is further reflected in niche-specific enzymatic profiles: surface communities express light-harvesting proteins, while deeper layers harbor enzymes for anaerobic respiration and degradation of complex organic matter (Noirungsee et al., 2024; Salwan and Sharma, 2022; Martínez-Espinosa, 2020; Wakai, 2019). Previous depth gradient studies in other salt flat systems, such as the “Sabkhas,” have found significant differences in the gene content involved in different components of biochemical cycles and secondary metabolite biosynthesis (Hazzouri et al., 2022); however, information from these niches remains scarce.

A deeper understanding of how hydrolytic enzymes stratify along depth gradients in hypersaline environments is crucial for clarifying mechanisms that govern organic matter turnover under extreme ionic and osmotic stress. Given their functional relevance and biotechnological potential, these enzymes provide valuable insights into how microbial communities adapt and specialize in response to depth-specific physicochemical constraints. Studying the functional stratification and enzymatic adaptation of microbial assemblages in the Uyuni Salt Flat can therefore reveal the evolutionary strategies that enable life to persist in extreme conditions and shed light on the mechanisms supporting community resilience and metabolic specialization (Gao et al., 2022; Tse and Ma, 2016; Somayaji et al., 2022).

Here, we aim to characterize microbial communities’ functional potential and enzymatic adaptations across different depth gradients within the Uyuni salt flat. By integrating metagenomic approaches with physicochemical assays, we aim to identify depth-specific functional traits and enzymatic profiles that support microbial survival and activity under extreme physicochemical conditions, thus contributing to a deeper understanding of microbial functionality in hypersaline systems.

## 2 Methods

### 2.1 Sampling design

**Figure 1:**
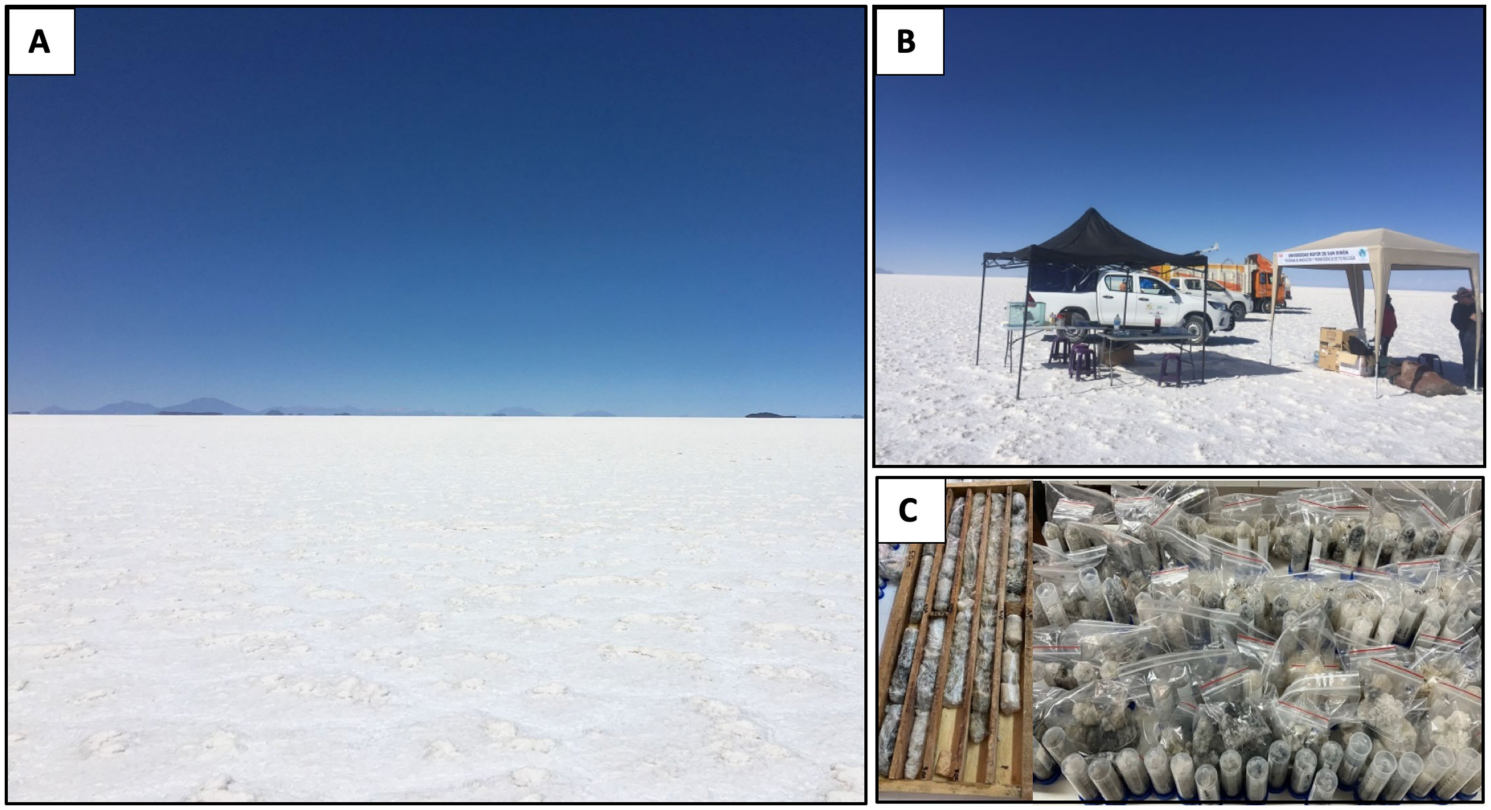
Sampling site at the Uyuni salt flat. **(A)** General view of the area; **(B)** Establishment of the drilling control center; **(C)** Drilling box and sub-sample collection.

Samples were obtained in the dry season of 2022 at Uyuni Salt Flat, Bolivia. Salt cores were obtained by rotary drilling with a diamond core in NQ diameter with a 90° inclination gradient to a depth of 8 meters at *S 20°14.974*″ *W 67° 30.*094″ after conducting site prospection and marking the designated location. The target depth of 8 meters was chosen based on prior geochemical reports that revealed clear physicochemical zonation below the halite crust (Fritz et al., 2004). Additionally, mechanical constraints related to drilling stability and core preservation limited sampling beyond this depth. Once the core samples were obtained, they were sealed in plastic, packed in wooden drilling boxes in 1-meter sections, and covered with salt from the drilling site as insulation. Core samples were stored and transported at ambient temperature and processed within 48 h. Upon arrival at the laboratory, the drilling boxes were opened, the plastic and salt removed, and sub-samples were taken for various analyses. To do this, the outer layer of the salt cores was discarded to avoid contamination with environmental microorganisms or cross-site contamination. Al sub-sampling work was carried out in a biosafety cabinet to ensure sterility. Pictures of the conducted sampling are shown in Figure 1.

### 2.2 Physicochemical analyses

Physicochemical analyses were conducted from four replicates per meter. Analyzed parameters included magnesium, manganese, nitrogen, sodium, potassium, chloride, sulfates, sulfites, total iron, lead, and organic matter. Magnesium, manganese, total iron, and lead were analyzed using atomic absorption (Koshino and Narukawa, 1993); nitrogen was measured using the Kjeldahl method (Tan et al., 2022); sodium, sulfates, and potassium concentrations were assessed using photometric method (Banerjee and Prasad, 2020); chloride and sulfides were analyzed using titrimetric method; organic matter was measured using gravimetric methods (Miyazawa et al., 2000; Sato et al., 2014).

### 2.3 DNA extraction and sequencing

Total DNA was extracted using the DNeasy® PowerSoil® Pro Kit (Qiagen), following the manufacturer’s protocol with the following modifications: salt samples were dissolved in DNase-free sterile water (UltraPure Distilled Water- Invitrogen) and subsequently filtered using 0.2 μm pore size polycarbonate membranes (Millipore) from which DNA was extracted. Additionally, an incubation step with proteinase K at 55 °C for 1 h and 3 freeze–thaw cycles (10 min per cycle), was included to improve DNA yield. Gel electrophoresis (1% agarose gel) was performed to evaluate DNA quality and purity, and DNA concentration was determined using a Qubit™ 4.0 fluorimeter (ThermoFisher Scientific).

DNA samples were then sent for library preparation and sequencing to the Advanced Genomics Core Lab of the Center for Biomedical Shared Resource at Vermont University for deep shotgun sequencing using the Singular Genomics System G4 platform to obtain 2 × 150 bp paired-end reads. For each metagenomic library, an expected sequencing depth of approximately 30 Gbp per sample was targeted, corresponding to an estimated average coverage of 10x based on predicted metagenome sizes (~30 Gb). Empirical coverage estimation was performed using Nonpareil (v.3.5.5) (Rodriguez-R et al., 2018), which estimates the average coverage of metagenome based on read redundancy.

The datasets generated in this study are publicly available in the Sequence Read Archive at https://www.ncbi.nlm.nih.gov/sra/PRJNA1311458

### 2.4 Trimming, quality control and bioinformatic processing

The raw sequence data was processed to remove sequence adapters, low-quality, un unpaired reads were trimmed using Fastp (v.0.23.4) (Chen et al., 2018; Beghini et al., 2021), with manual specification of Singular Genomics System G4 platform adapter sequences, trimming of the first 10 bases from the 5′ end of each read, quality trimming at both ends with a minimum mean quality threshold of Q20, and a minimum length cut-off of 50 bp. Human sequences were removed using KneadData (v0.12.2, available in http://github.com/biobakery/kneaddata) using the human genome hg38 as a reference. Functional metagenomic profiles were then generated using HUMAnN 3 (v3.9) (Beghini et al., 2021) using default parameters. Briefly, metagenomic reads were first taxonomically profiled using MetaPhlAn 3 (Beghini et al., 2021) to identify community composition. A tiered alignment strategy was then applied; reads were initially mapped against custom pangenomes using Bowtie2 (Langmead and Salzberg, 2012) for species-resolved functional annotation, and unmapped reads were subsequently aligned against the UniRef90 (Suzek et al., 2007) protein database using DIAMOND (Buchfink et al., 2015) to maximize gene family detection. The resulting enzyme abundances were reported in units of reads per kilobase (RPK), which normalize read counts by gene length to allow comparisons across gene families. Theses normalized profiles were subsequently aggregated by enzyme category and used for downstream statistical analyses and visualization.

Enzymes were manually classified into hydrolytic and non-hydrolytic groups based on EC numbers and functional roles, with emphasis on ecological relevance in hypersaline sediments. Hydrolytic enzymes corresponded predominantly to EC class 3 (hydrolases), which catalyze the cleavage of bonds via water. Although peroxidases belong formally to EC class 1 (oxidoreductases), they were included in the hydrolytic group due to their central role in the oxidative breakdown of complex organic matter such as necromass and microbial exopolymers, a function ecologically analogous to hydrolysis in carbon cycling under poly-extreme conditions (Merino et al., 2020; Li et al., 2024; Stursova and Sinsabaugh, 2008).

### 2.5 Statistical analysis

Spearman’s rank correlation analysis was performed to assess the association between physicochemical parameters and changes in the relative abundance of microbial enzymes. Prior to analysis, all physicochemical variables were standardized using z-score normalization to facilitate comparability across different scales. Correlation matrices and corresponding *p*-values were computed using the rcorr() function from the Hmisc package in R (v.5.1) (Harrell Jr, 2025). Correlations were visualized using the corrplot package (v.0.95) (Wei and Simko, 2024) through a heatmap displaying statistically significant correlations (*p* < 0.05). Enzymes with ecological relevance—amylases (EC 3.2.1.-), cellulases (EC 3.2.1.-), glucosidases (EC 3.2.1.-), lipases (EC 3.1.1.-), peptidases (EC 3.4.-.-), peroxidases (EC 1.11.1.-), proteases (EC 3.4.-.-), and xylanases (EC 3.2.1.-)—were grouped and ordered to enhance biological interpretability. Enzymatic structure patterns were explored through hierarchical clustering based on Bray–Curtis dissimilarity.

Normality and homogeneity of variances were assessed using the Shapiro–Wilk and Levene’s tests, respectively. When these assumptions were met, analysis of variance (ANOVA) was applied; otherwise, non-parametric comparisons were conducted using the Mann–Whitney U test. All statistical analyses were performed using R version 4.3.2 (R Development Core Team, 2020, Vienna, Austria). Differences were considered statistically significant at *p* < 0.05. Al statistics were performed using the R package vegan (v.2.6–4).

## 3 Results

### 3.1 Changes in the physicochemical variables with depth

**Figure 2:**
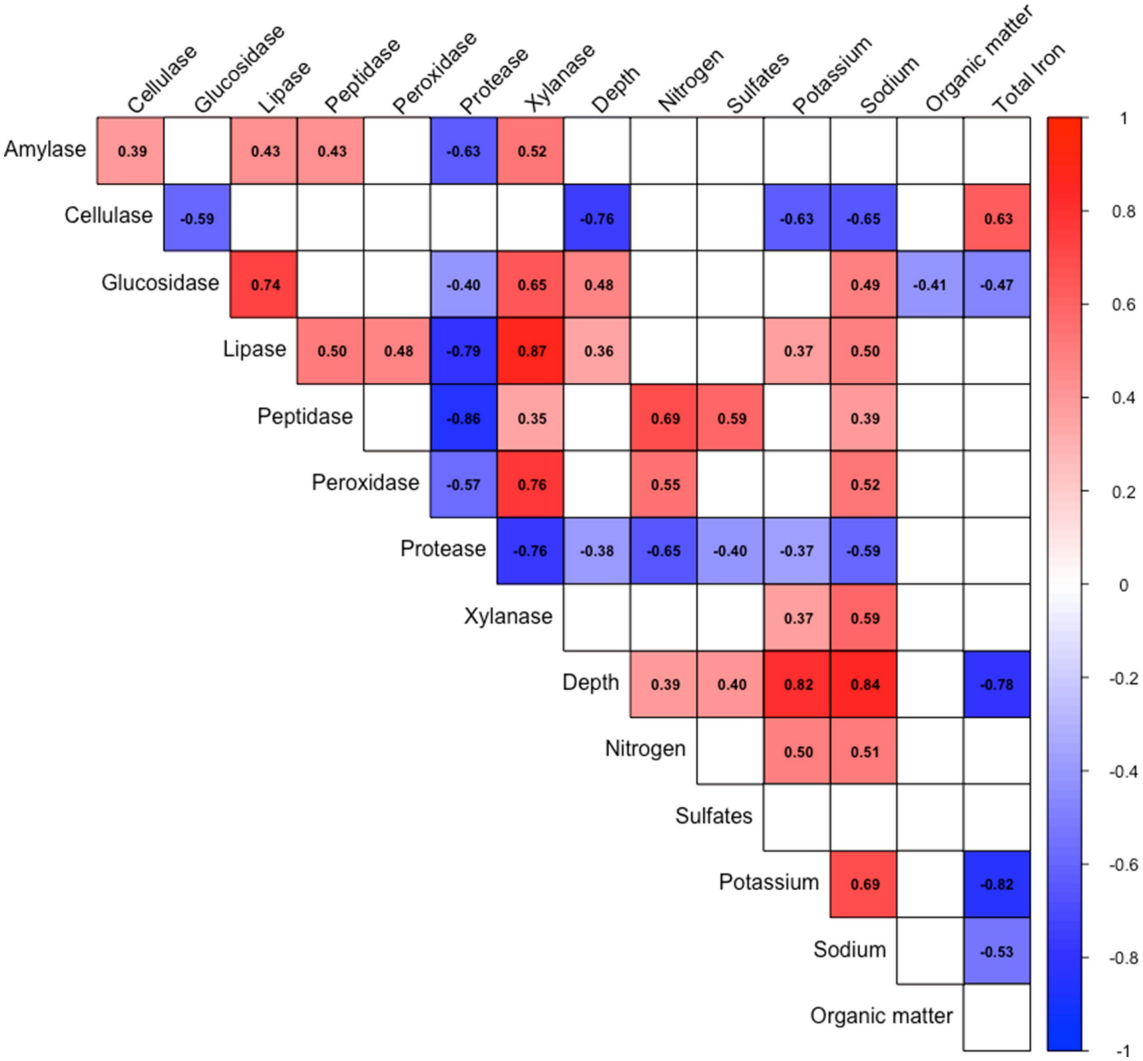
Correlation matrix between abiotic factors and hydrolytic enzyme abundance. Only statistically significant correlations (*p* < 0.05) are shown. Positive correlations are indicated in red, while negative correlations are shown in blue. Color intensity reflects the strength of the correlation. Correlation values are based on Spearman’s rank correlation coefficients.

Most environmental parameters changed significantly across different depths (Table 1). Magnesium, manganese, nitrogen, potassium, sodium, and sulfate concentrations were positively correlated with depth increase, while chlorides, total iron, and lead exhibited a negative correlation with depth. Regarding enzyme abundance, cellulases and proteases exhibited negative correlations with depth, while glucosidases and lipases were positively correlated with depth increase (Figure 2).

**Table 1:** Physicochemical variables across 8-meter depth gradient.

	M1	M2	M3	M4	M5	M6	M7	M8	*p*
Nitrogen	3.39 ± 0.03	4.33 ± 0.02	6.95 ± 0.14	4.67 ± 0.02	4.33 ± 0.01	4.45 ± 0.01	4.03 ± 0.05	8.88 ± 0.02	8.7 e-5
Chlorides	574.79 ± 0.1	550.23 ± 0.08	581.34 ± 0.05	580.85 ± 0–05	556.25 ± 0.05	564.75 ± 0.01	512.69 ± 0.02	539.92 ± 0.01	7.5 e-5
Nitrates	15 ± 0.07	15 ± 0.11	17.1 ± 0.11	19.9 ± 0.11	14 ± 0.11	14.03 ± 0.09	15 ± 0.15	46.6 ± 0.25	1.5 e-4
Sulfates	4.37 ± 0.01	18.85 ± 0.01	5.03 ± 0.01	9.6 ± 0.01	6.37 ± 0.01	7.66 ± 0.01	13.9 ± 0.01	12.22 ± 0.01	7.5 e-5
Calcium	20.03 ± 0.01	133.2 ± 0.08	8.51 ± 0.01	12.17 ± 0.01	7.08 ± 0.02	3.99 ± 0.01	25.53 ± 0.01	354.49 ± 0.01	7.5 e-5
Magnesium	1.35 ± 0.01	0.73 ± 0	1.02 ± 0	1.32 ± 0.01	1.3 ± 0.01	1.19 ± 0.01	1.64 ± 0.01	4.11 ± 0.01	9.5 e-5
Potassium	1.71 ± 0.01	1.69 ± 0.02	2.16 ± 0.01	1.81 ± 0.01	2.2 ± 0.01	2.18 ± 0.01	2.17 ± 0.02	8.45 ± 0.02	2.6 e-4
Sodium	384.46 ± 0.75	392.49 ± 1.16	392.4 ± 0.98	410.02 ± 1.84	412.8 ± 2.03	410.79 ± 1.18	408.39 ± 0.72	418.86 ± 0.53	2.9 e-4
Organic mater	0.56 ± 0	1.1 ± 0	1.08 ± 0	0.76 ± 0	1.07 ± 0	1.24 ± 0.04	0.8 ± 0	0.86 ± 0	8.7 e-5
Sulfides	73.8 ± 0.74	58.65 ± 1.04	84.7 ± 1.3	56.8 ± 1.24	81.5 ± 1.36	58.6 ± 2.08	60.5 ± 1.38	62.1 ± 2.22	7.6 e-4
Total Iron	56.9 ± 0.7	64.37 ± 0.17	44.81 ± 0.14	70.54 ± 0.54	30.32 ± 0.99	42.18 ± 1.13	23.82 ± 1.58	21.39 ± 0.67	9.3 e-5
Lead	18.95 ± 0.14	20.47 ± 0.19	19.99 ± 0.39	18.86 ± 0.2	20.7 ± 0.64	17.36 ± 0.19	13.56 ± 0.25	9.53 ± 0.14	3.4 e-4
Manganese	15.87 ± 0.09	24.88 ± 0.84	9.28 ± 0.3	14.79 ± 0.34	17.31 ± 0.53	NA	27.65 ± 0.43	38.84 ± 0.34	1.9 e-4

### 3.2 Sequencing depth variability and dataset overview

Between 25 and 132 million pared-end reads were obtained per sample, corresponding to a raw sequencing depth ranging from approximately 2.5 Gbp to 18 Gbp across the eight metagenomes. After quality filtering and trimming, as well as removal of reads identified as potential human DNA contamination, between ~ 22 and 115 million pared-end reads per sample were retained, resulting in sequencing depths ranging from 2.1 Gb to 15.2 Gb. This variation in sequencing depth was largely attributed to the low DNA concentrations that were obtained from several sediment layers. Such limited yields are commonly observed when working with hypersaline sediments characterized by low microbial biomass and the presence of chemical inhibitors, such as manganese and other chaotropic salts, which may interfere with DNA extraction efficiency and downstream library preparation.

To further evaluate the effective sequencing coverage achieved, Nonpareil analysis was performed on all datasets. The results showed estimate coverage values ranging from 91.9 to 99.9%, with most samples exceeding 97% coverage indicating that despite variability in sequencing depth, the sequencing effort was sufficient to capture the majority of the microbial community diversity and functional potential present in each sample.

### 3.3 Functional and family-level diversity patterns

**Figure 3:**
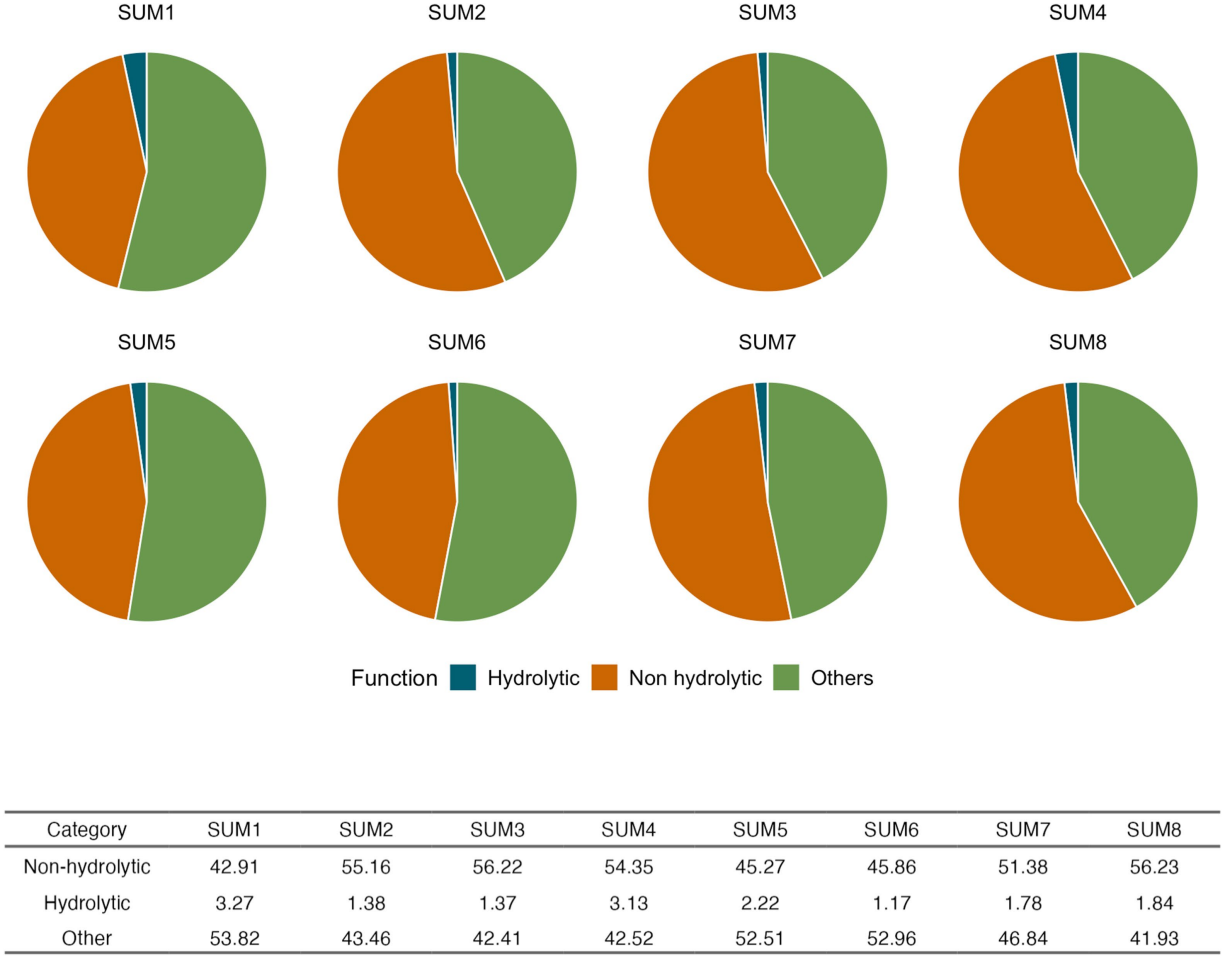
Functional profiles at a depth gradient (SUM1-SUM8). Functions were classified as hydrolytic enzymes (blue), non-hydrolytic enzymes (orange), and “other” functions (green). Significant differences were observed (KW *p* = 0.0019) indicating depth-related shifts in overall functional composition. Mann–Whitney pairwise comparisons showed significant differences between depths 1–2, 1–3, 1–8, 3–5, 5–8, and 6–8.

Functional profiles classified as hydrolytic enzymes (blue), non-hydrolytic enzymes (orange), and other non-enzymatic functions (green) exhibited clear depth-dependent variability across the eight-meter core (Figure 3). Nonhydrolytic enzymes were the dominant functional group in all layers, particularly enriched in deeper sediments. Functions grouped as “Other” in green, represented the second most abundant category. Lastly, hydrolytic enzymes (blue), typically involved in the breakdown of complex organic polymers, were the least abundant across all depths. A Kruskal-Wallis test confirmed significant shifts in overall functional composition among depths (*p* = 0.0019), and subsequent Mann–Whitney U tests pairwise comparisons for all depth combinations reinforced a pronounced functional stratification along the hypersaline depth gradient of the Uyuni Salt Flat.

Family-level taxonomic composition also displayed clear depth-dependent patterns across the eight-meter core (Supplementary Figure S1). Acetobacteraceae and Acidiferrobacteraceae exhibited significant positive correlations with depth, suggesting an ecological preference for deeper, more extreme conditions. In contrast, several taxa including Desulfohalobiaceae, Halobacterales, Halobacteriaceae, Propionibacteriaceae, Pseudomonadaceae, Rhodobacteraceae, and Woesearchaeales showed significant negative correlations with depth, indicating a dominance in shallower layers. These opposing trends highlight a pronounced taxonomic stratification along the hypersaline gradient, consistent with environmental filtering across depth.

### 3.4 Depth-dependent patterns of hydrolytic enzymes

**Figure 4:**
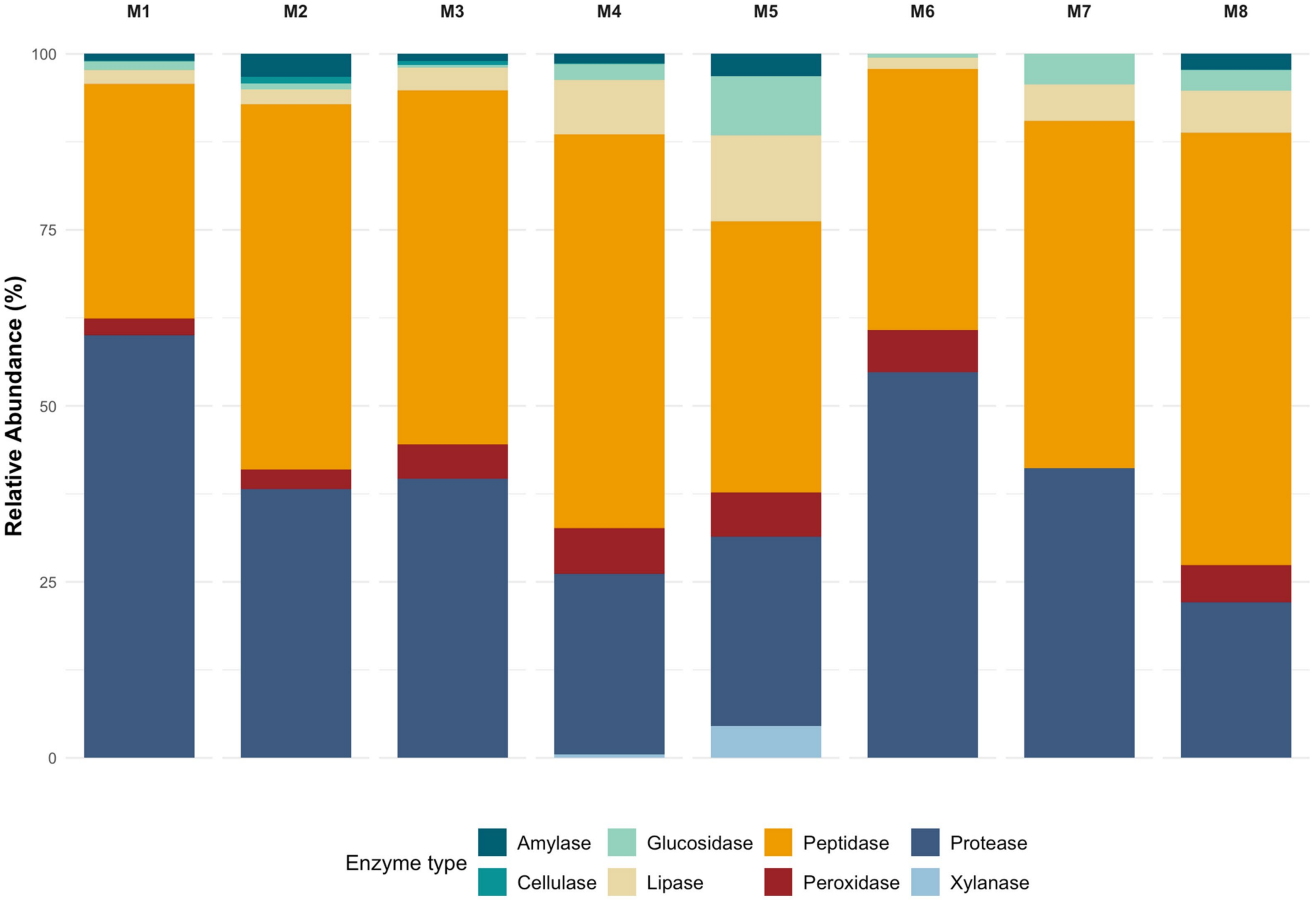
Depth dependent patterns for hydrolytic enzymes. Depth-dependent patterns of enzyme distribution were evidenced for peptidases, glucosidases, lipases, and proteases (KW *p* = 0.038). Although no overall significance was found for cellulases and xylanases, both showed significant pairwise differences. Amylases and peroxidases showed a similar depth-related trend.

When examining the relative abundance of hydrolytic enzymes—amylases, cellulases, glucosidases, lipases, peptidases, peroxidases, proteases and xylanases—along the hypersaline depth gradient of the Uyuni salt flat (Figure 4). Depth-dependent patterns of enzyme distribution are evident, with significant differences detected for several enzyme classes. Specifically, peptidases, glucosidases, lipases, and proteases exhibited statistically significant variation with depth, as indicated by the Kruskal-Wallis test (*p* = 0.038), and all Mann–Whitney pairwise tests were also statistically significant. Cellulases and xylanases did not achieve global significance (*p* = 0.068 and non-significant, respectively), yet both displayed statistically meaningful pairwise differences, suggesting localized fluctuations despite intermittent absence in certain layers. Amylases (*p* = 0.056) and peroxidases (*p* = 0.046) followed a similar pattern of marginal global *p*-values paired with consistent significance across all depth contrasts, indicating fine-scale stratification of these enzyme classes.

### 3.5 Subtype-level variability among hydrolytic enzymes

**Figure 5:**
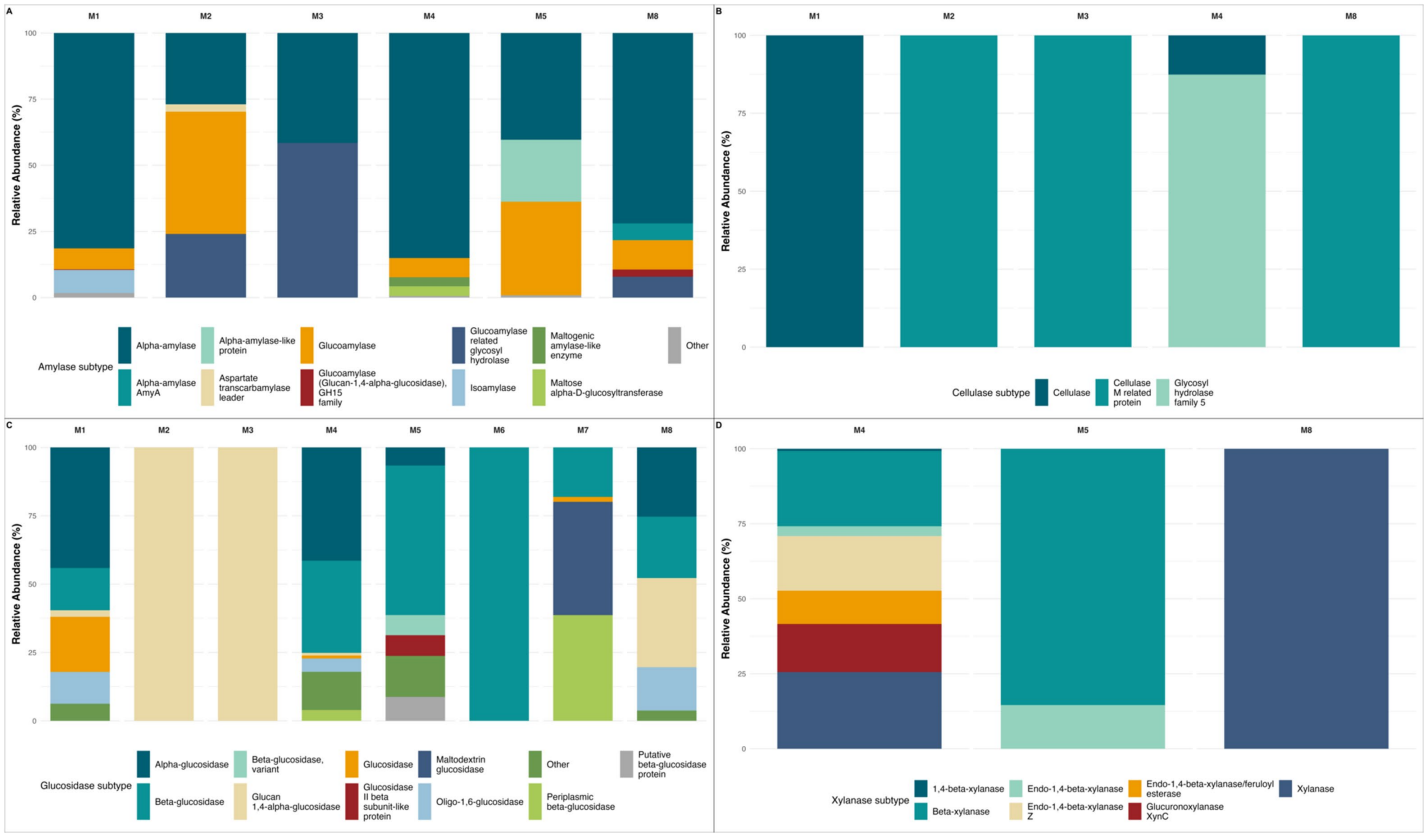
Polisacharide-degrading enzymes subtype variability with depth. **(A)** Relative abundance for amylolytic enzymes, alpha-amylase, glucoamylase, and maltose alpha-D-glucosyltransferase showed significant depth-related differences (KW *p* < 0.05); **(B)** Relative abundance for cellulolytic enzymes, generic “cellulase” showed a significant depth-dependent distribution (KW *p* = 0.000172); **(C)** Relative abundance for glycosidic enzymes, all dominant subtypes showed a depth related pattern (KW *p* < 0.05); **(D)** Relative abundance for xylanases, only beta-xylanase exhibited significant variation with depth. Missing depths in some panels reflect samples where no reads were assigned to the corresponding enzyme families after quality filtering.

The relative abundance of enzyme subtypes was analyzed to obtain a comprehensive analysis of the abundance of the different hydrolytic enzymes. Figure 5A shows the relative abundance of amylase subtypes. Significant differences in amylase-related enzyme abundance were observed across layers. Alpha-amylase (EC 3.2.1.1), glucoamylase (EC 3.2.1.3), glucoamylase related glycosyl hydrolase (EC 3.2.1.3), and “other” amylases showed significant differences (*p* < 0.05) among depths. At pairwise comparisons, alpha-amylase displayed significant differences between meters 1–3, 1–4, and 3–4, suggesting strong differentiation in abundance at those depths. Glucoamylase also exhibited significant differences in depth-related abundance, especially between meters 1–2, 2–5, and 5–8. These findings indicate depth-related variation in the distribution of several amylolytic enzymes within the sediment profile.

Among the cellulolytic enzymes evaluated (Figure 5B), only the general “cellulase” category showed statistically significant differences in relative abundance across the depth gradient. Pairwise comparisons using the Mann–Whitney U test revealed a marginally significant difference in cellulase abundance between meters 1 and 4. Regarding glucosidases (Figure 5C), alpha-glucosidase (EC 3.2.1.20) and beta-glucosidase (EC 3.2.1.21) showed the most pronounced differences, followed by enzymes such as oligo-1,6-glucosidase (EC 3.2.1.10). Pairwise comparisons identified statistically significant differences for beta-glucosidase between meters 4–5, 4–6 and 5–6. Similarly, for alpha-glucosidase, significant differences were observed between meters 4–1, 5, and 8.

**Figure 6:**
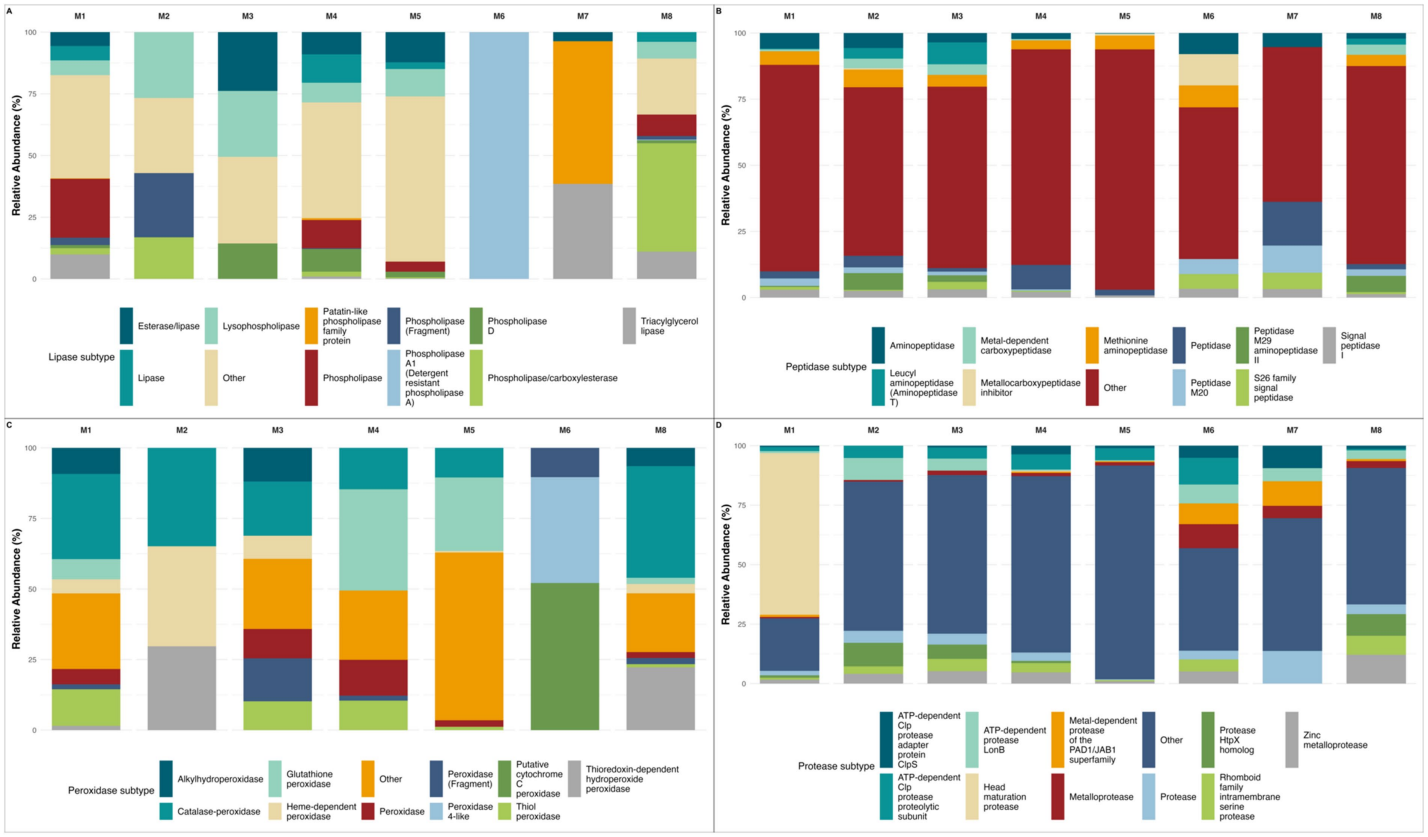
Protein, lipid, and oxidative-degrading enzymes subtype variability with depth. **(A)** Relative abundance for lipolytic enzymes, all major subtypes showed significant differences except Phospholipase A1 and phospholipase fragment; **(B)** Relative abundance for peptidic enzymes, all dominant subtypes displayed marked depth-related variation; **(C)** Relative abundance for peroxidatic enzymes, all the most abundant subtypes exhibited a depth-dependent pattern; **(D)** Relative abundance for proteolytic enzymes, all dominant subtypes showed significant stratification; Missing depths in some panels reflect samples where no reads were assigned to the corresponding enzyme families after quality filtering.

Among lipolytic enzymes (Figure 5D), all of the most abundant lipase subtypes exhibited statistically significant differences across layers, with the only exception of phospholipase A1 (EC 3.1.1.32) (detergent-resistant phospholipase A), which exhibited borderline significative differences. On pairwise comparisons, only lysophospholipase (EC 3.1.1.5) abundance exhibited significant difference between meters 2–4, and 3–4. Regarding peptidases (Figure 6A), all the most abundant subtypes exhibited statistically significant differences across the depth profile. On pairwise comparisons, aminopeptidase (EC 3.4.11.-), S26 signal peptidase (EC 3.4.21.89), generic “peptidase,” and signal peptidase I (EC 3.4.21.89) abundances exhibited significant differences among most depths. Among peroxidases (Figure 6B), all enzyme subtypes exhibited significant differences across layers. When comparing pairwise, catalase-peroxidase abundance differed between most depths, whereas other subtypes appeared to be more evenly distributed along the gradient, suggesting that significant Kruskal-Wallis results were driven by abundance differences between only a few specific depths. The same tendency was observed for proteolytic enzymes (Figure 6C) where all protease subtypes exhibited significative differences along the depth gradient, but only ATP-dependent Clp protease (EC 2.4.21.92), ATP-dependent protease LonB (EC 3.4.21.109), and Zinc metalloprotease (EC 3.4.24.-) abundances exhibited significant differences among most depths. Regarding xylanases (Figure 6D), statistical analyses did not reveal significant differences across depths. Nonetheless, clear patterns in enzyme abundance and subtype diversity are evident. This apparent discrepancy may be attributed to the absence of most subtypes at several depths, which likely reduced the statistical power to detect significant differences despite the observable variation.

### 3.6 Non-hydrolytic enzyme subtype stratification

**Figure 7:**
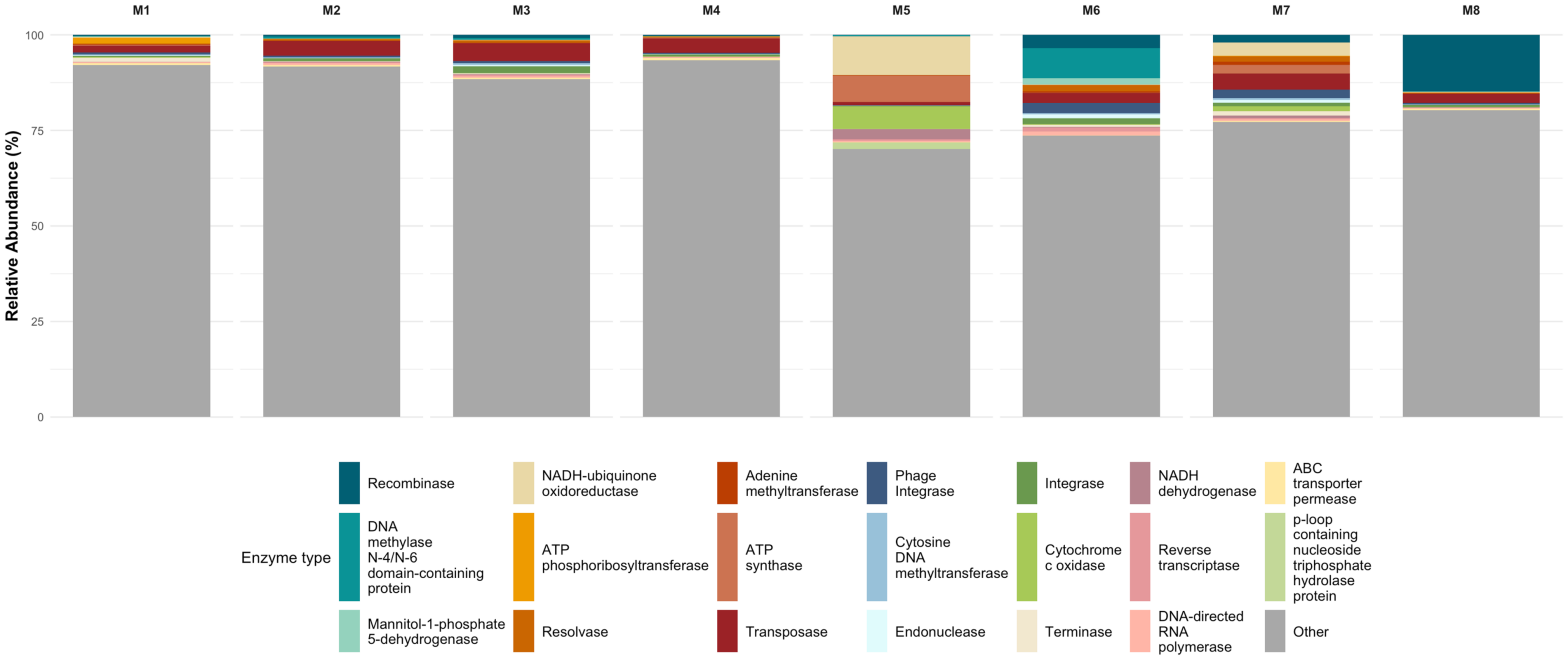
Non-hydrolytic enzyme abundance. Relative abundance of non-hydrolytic enzymes exhibited a clear depth-dependent stratification (KW *p* < 0.05), with the exception of cytosine DNA methyltransferase (*p* = 0.114), DNA methylase N4 (*p* = 0.379), and adenine methyltransferase (*p* = 0.721). Pairwise Mann Whitney test further supported this pattern, revealing significant differences across most depths.

Analysis of non-hydrolytic enzyme subtypes (Figure 7), demonstrated that, except for DNA methylase N-4 (EC 2.1.1.113), adenine methyltransferase (EC 2.1.1.72), and cytosine DNA methyltransferase (EC 2.1.1.37), all major enzymes in this group exhibited significant shifts along the gradient. Notably, recombinases, transposases, and integrases showed increased relative abundance in the deepest samples, suggesting a greater prevalence of horizontal gene transfer and genomic rearrangement under extreme environmental stress. Additionally, energy-related enzymes such as NADH–ubiquinone oxidoreductase, ATP synthase, and cytochrome C oxidase were more abundant in intermediate and deep samples, potentially reflecting metabolic adjustments to cope with energetic demands in high ionic strength conditions. The enrichment of ABC transporter permeases and P-loop NTPases may also indicate an increased requirement for solute transport and osmoregulation mechanisms in these environments.

Interestingly, the consistent presence of reverse transcriptase and phage integrase across samples could imply ongoing viral activity or lysogenic cycles, possibly contributing to microbial turnover or horizontal gene flow.

### 3.7 Functional stratification across the depth gradient

**Figure 8:**
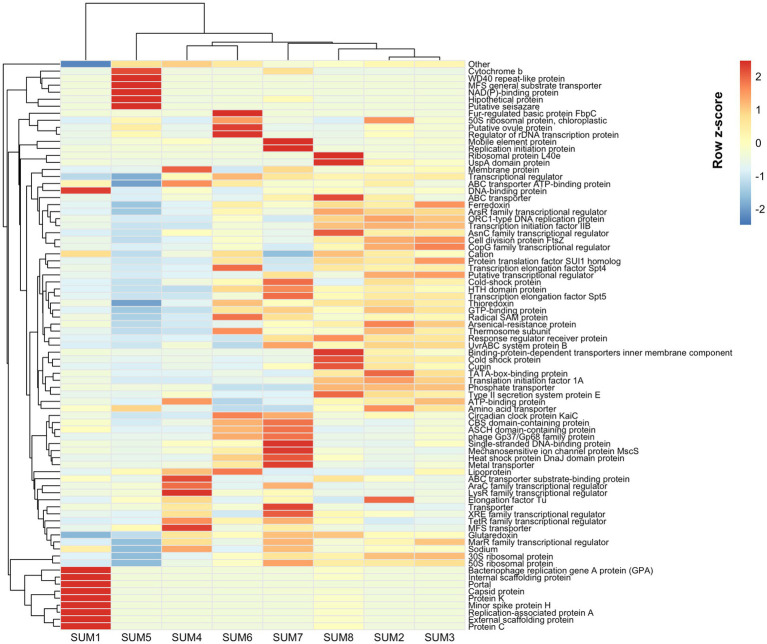
Functional stratification across depths. Clustered row-scaled relative abundances revealed four distinct functional horizons. Surface layers were enriched in phage-related proteins; mid-depths in membrane transport and osmotic stress-related factors; deeper layers in housekeeping functions.

Row-scaled relative abundance variabilities, clustered across proteins and the eight core depths (Figure 8), revealed four functionally coherent horizons. In the surface (SUM1), phage-associated proteins such as portal, capsid, scaffolding, and replication factors were uniquely over-represented. The mid-depth transition zone (SUM4-SUM5) was marked by pronounced enrichments of membrane transport systems (MFS, ABC, amino acid, and phosphate transporter), NAD(P)-binding domains and osmoprotectant-related factors, consistent with peak osmotic stress and active solute uptake. In contrast, deeper layers (SUM6-SUM8) clustered together due to their high relative variability in core “housekeeping” functions—ribosomal proteins (30S, 50S), replication initiation factors (ORC1), transcriptional and elongation machinery, and DNA repair complexes (UvrABC). The intermediate layers (SUM2 and SUM3) displayed near-baseline variability across most functional categories.

## 4 Discussion

The Uyuni Salt Flat is an endorheic system composed of alternating salt and clay layers reaching depths of more than 121 meters, each with a distinct geochemical signature. Its nearly 50,000-year climatic history has shaped cycles of evaporation and recharge with salts such as saturated NaCl, MgCl_2_, and LiCl, creating a osmotropic/chaotropic environment that selects for extremophiles with osmoregulatory mechanisms (Haferburg et al., 2017). The observed inverse relationship between chloride/iron and depth (Figure 2) aligns with previous reports of lithium-rich brines in Uyuni salt flat. The depth-dependent chaotropicity, driven by Mg^2+^ and lithium gradients, creates distinct microbial survival strategies across the vertical profile. Moreover, the positive Mg^2+^ depth correlation aligns with chaotropic stress intensification in deeper layers, where Mg^2+^ concentrations reach levels known to disrupt cellular macromolecules and membrane integrity (Rubin et al., 2017). This chaotropic likely drives the observed enrichment of non-hydrolytic enzymes in deeper layers (Figure 3), as microorganisms prioritize osmoregulation over polymer degradation under extreme ionic stress (Rubin et al., 2017).

Episodic rainfall events drive the annual deposition of salts, redistribute sediments, flatten the salt crust, and renew the supply of organic and mineral inputs to the surface. The combination of high salinity and acidic pH makes Uyuni a polyextreme habitat, where microorganisms produce osmoprotectants and extracellular biopolymers to survive (Chambi et al., 2021). In addition, drastic fluctuations in temperature, humidity, and UV radiation increase oxidative stress, demanding specialized enzymatic systems for protection and repair (Martínez et al., 2021). During the wet season, temporary surface water bodies moderate local salinity and provide organic matter to the underlying sediment (Williams and Vengosh, 2025).

Wet-dry cycles induce nutrient pulses, and rapid salinity shifts, transiently enhancing hydrolytic pathways such as amylases and glucosidases in surface strata (Cubillos et al., 2019). Indeed, all major amylase subtypes varied significantly with depth suggesting episodic inputs of starch-rich organic matter deposited during wet seasons, which are progressively degraded by distinct amylolytic consortia as the material is buried, a process also observed in other hypersaline ecosystems (Dang and Lovell, 2016). This season variability may also account for the observed positive correlations between glucosidase/lipase activity and depth, reflecting specialized heterotrophic niches in these deeper anaerobic zones, indicating a pivot to simpler substrates such as microbial necromass and soluble oligosaccharides, consistent with reported sulfate-reducer and methanogen communities in hypersaline sediments (Martínez et al., 2021; Varrella et al., 2020). Lipolytic enzymes exhibited one of the strongest depth gradients; their enrichment of lipases in mid to deep sediments suggests a transition to lipid necromass as the predominant carbon source once carbohydrates are depleted (Guo et al., 2019).

Conversely, the decrease in protease and cellulase activities with depth may be explained by a reduction in fresh organic matter and complex polysaccharides, while a higher relative abundance of these enzymes in superficial layers likely reflect the exploitation of recent allochthonous inputs such as algal exopolymers and atmospheric dust (Martínez et al., 2021; Amoozegar et al., 2017). Patchy zones of peroxidases and amylase abundance, detected by marginal yet significant *p*-values may indicate fine-scale adjustments to localized geochemical gradients such as small fluctuations in edaphic factors that may trigger peroxidase induction to quench reactive oxygen species (Martínez et al., 2021). While peroxidase families showed global stratification, catalase-peroxidase was particularly differentiated abundance along most depths; other peroxidase subtypes peaked at distinct horizons, underscoring that antioxidant defenses are a core strategy for coping with micro-gradients of oxidative stress driven by high solar radiation and redox cycling. Such micro-zones likely foster specialized consortia that occur transiently, maintaining overall community resilience under specific environmental stressors (Martínez-Alvarez et al., 2023). Despite non-significant global trends on cellulase and xylanase abundance, their consistent pairwise differences may suggest episodic hotspots of complex-carbohydrate degradation, linked to localized detritus deposition (Martínez-Alvarez et al., 2023), similar observations have been reported in the Great Salt Lake, where cellulose degrading taxa appear only after rare allochthonous plant detritus events (Oren, 2013).

Major peptidase and protease subtypes exhibited significant depth-dependent differences, with aminopeptidase, S26 signal peptidase, and ATP-dependent Clp proteases showing widespread variation across most depths. These enzymes facilitate protein turnover and stress response, reflecting microbial adaptation to nutrient gradients and environmental stressors such as high salinity, UV radiation, and chaotropic ions (De Castro et al., 2006). The stratified functional organization exhibited in the depth gradient enables nutrient cycling under extreme conditions, maintaining microbial productivity across the system (Cubillos et al., 2019). Halotolerant and antioxidant pathways provide resilience to precipitation pulses, seasonal transitions, and redox fluctuations, ensuring community persistence beyond extreme events (Paquis et al., 2023). Altogether, the depth-resolved analysis of amylolytic, cellulolytic, glycosidic, lipolytic, peptidic, peroxidatic, and proteolytic enzyme subtypes demonstrate clear stratification of hydrolytic potential within the Uyuni salt flat sediment column.

Additionally, the coupling between enzymatic activities and taxonomic shifts suggests that depth-resolved microbial communities in the Uyuni Salt Flat are both functionally and taxonomically organized in response to gradients of organic matter, oxygen, and osmotic stress. The observed taxonomic distribution, such as those of Acetobacteraceae, Acidiferrobacteraceae, Halobacterales, Halobacteriaceae, Haloferacaceae, Propionibacteriaceae, Pseudomonadaceae, Rohobacteraceae, and Woesearchaeales, reflect the presence of specialist consortia that partition resources and occupy niches shaped by the availability of fresh detritus at the surface and more recalcitrant, microbially-derived substrates at depth. This ecological stratification enables resilience in presence of fluctuating redox conditions, episodic inputs of organic matter, and sustained poly-extreme stressors, consistent with patterns observed in other hypersaline and deep-surface environments (Merlino et al., 2018; Drissi Kaitouni et al., 2020; Weingarten et al., 2020).

When analyzing non-hydrolytic enzyme subtypes, these exhibited significant shifts along the depth gradient, except for DNA methylases, indicating dynamic regulation of DNA modification and repair processes tailored to depth-specific environmental pressures such as UV exposure and ionic stress. The enrichment of phage-associated proteins such as portal, capsid, scaffolding and replication factors in the surface layer aligns with the high viral predation pressures documented in UV-exposed hypersaline environments like the Uyuni Salt Flat. High viral-to-prokaryote ratio have been reported in similar high-altitude wetlands, indicating intense virus-host interactions that influence microbial community dynamics and turnover at the surface (Eissler et al., 2022). The extreme UV radiation at the surface promotes viral activity and lytic cycles, which in turn shape microbial diversity and drive horizontal gene transfer, contributing to rapid microbial adaptation in this polyextreme habitat (Eissler et al., 2022; Breitbart et al., 2018).

The pronounced enrichment of membrane transport systems—including major facilitator superfamily (MFS), ATP-binding cassette (ABC) transporters, amino acid, and phosphate transporters—reflects active osmolyte uptake strategies under peak ionic and chaotropic stress, particularly from magnesium and lithium ions (Rubin et al., 2017; Haferburg et al., 2017) in the mid-depth horizon. In the deepest layers, the high variability and relative abundance of core “housekeeping” functions—such as ribosomal proteins (30S, 50S), replication initiation factors (ORC1), transcriptional and elongation machinery, and DNA repair complexes (UvrABC)—indicate adaptations to stable but nutrient-poor, anoxic brines (Oren, 2024). Similar functional profiles have been reported in deep hypersaline basins and subsurface brines, where microbial communities rely on efficient protein synthesis and DNA repair to persist in oligotrophic extreme environments (Ramos-Barbero et al., 2019).

Layered enzyme functions underpin multi-trophic cycles of carbon, nitrogen and sulfur: surface hydrolysis yields soluble substrates for mid-depth fermenters, whose byproducts power deep-layer lipid degraders and sulfur respiring guilds. This biogeochemical assembly line sustains microbial productivity despite extremes of salinity, osmotic stress, and other variables. Understanding these ecological dynamics is key for the bioprospecting of enzymes with biotechnological potential. From a biotechnological perspective, mining deep-core lipases, peroxidases and glucosidases offer catalysts stable in high-salt, low-water-productivity processes, ideal for saline wastewater bioremediation, halogenated-compound synthesis and bioenergy production under harsh industrial conditions (Martínez et al., 2021).

Our findings are consistent with previous observations in other salt flat ecosystems, where strong functional and taxonomic stratification emerges in response to steep salinity and nutrient gradients. Across diverse settings, microbial communities have been shown to exhibit high taxonomic novelty, functional redundancy, and metabolic versatility, supported by stress-tolerance traits, arsenic and oxidative stress adaptations, and horizontal gene transfer (Veloso et al., 2023; Martínez-Alvarez et al., 2023; Hazzouri et al., 2022). These characteristics enable microbial consortia to sustain carbon, nitrogen, and sulfur cycling despite the harsh poly-extreme conditions of these environments. Building on these insights, our study provides a depth-resolved perspective of hydrolytic enzyme distributions, illustrating how resource partitioning and niche specialization underpin microbial resilience in hypersaline sediment columns.

Overall, our study contributes to elucidating how depth-related geochemical gradients in the Uyuni salt flat drive a finely stratified distribution of both hydrolytic and non-hydrolytic enzymes across depth, revealing distinct microbial niches adapted to depth specific stressors and nutrient availability. Although sequencing depth varied among samples due to low DNA yields typical of hypersaline sediments with low microbial biomass and presence of chemical inhibitors such as manganese and chaotropic salts, the data obtained were sufficient to recover dominant functional patterns and enzymatic stratification across the gradient.

Hydrolytic enzymes were selected as the primary focus of this study because of their central ecological role in organic carbon turnover and nutrient cycling, as well as their potential applications under industrially relevant high-salt conditions. These insights not only advance our understanding of microbial life in polyextreme habitats but also highlight the vast biotechnological potential of extremozymes for biotechnological and remediation applications. Beyond revealing clear depth-dependent enzymatic stratification, our findings underscore the need for more comprehensive functional profiling to fully capture the complexity and adaptive strategies of microbial communities inhabiting poly-extreme environments. As an initial exploration of enzymatic potential in the Uyuni Salt Flat, this study lays the ground for future integrative analyses to link microbial taxa to their functional roles in deep-soil multi-extreme environments. Ultimately, this work establishes a framework for exploring functional stratification in other extreme systems.

## 5 Conclusion

These functional layers in the Uyuni Salt Flat reflect microbial stratification patterns observed in other extreme ecosystems, where surface layers are shaped by viral predation and UV stress, mid-depths by osmotic and chaotropic pressures requiring active transport and osmoprotection, and deep layers by adaptations to nutrient limitation and genomic maintenance. This vertical functional stratification emphasized the intricate relationship between environmental gradients and microbial ecological strategies.

## Data Availability

The datasets generated and/or analyzed during the current study are available at https://www.ncbi.nlm.nih.gov/sra/PRJNA1311458.

## Supplementary material

Supplementary Figure S1 Family-level taxonomy. Relative abundance of 20 most abundant families across samples.
