# Supplementary figures and images for: Functional stratification and enzymatic arrangement in microbial communities across a hypersaline depth gradient
Source: Front Microbiol. 2025 Sep 17;16:1624058. doi: 10.3389/fmicb.2025.1624058 (PMC12486415; doi:10.3389/fmicb.2025.1624058)

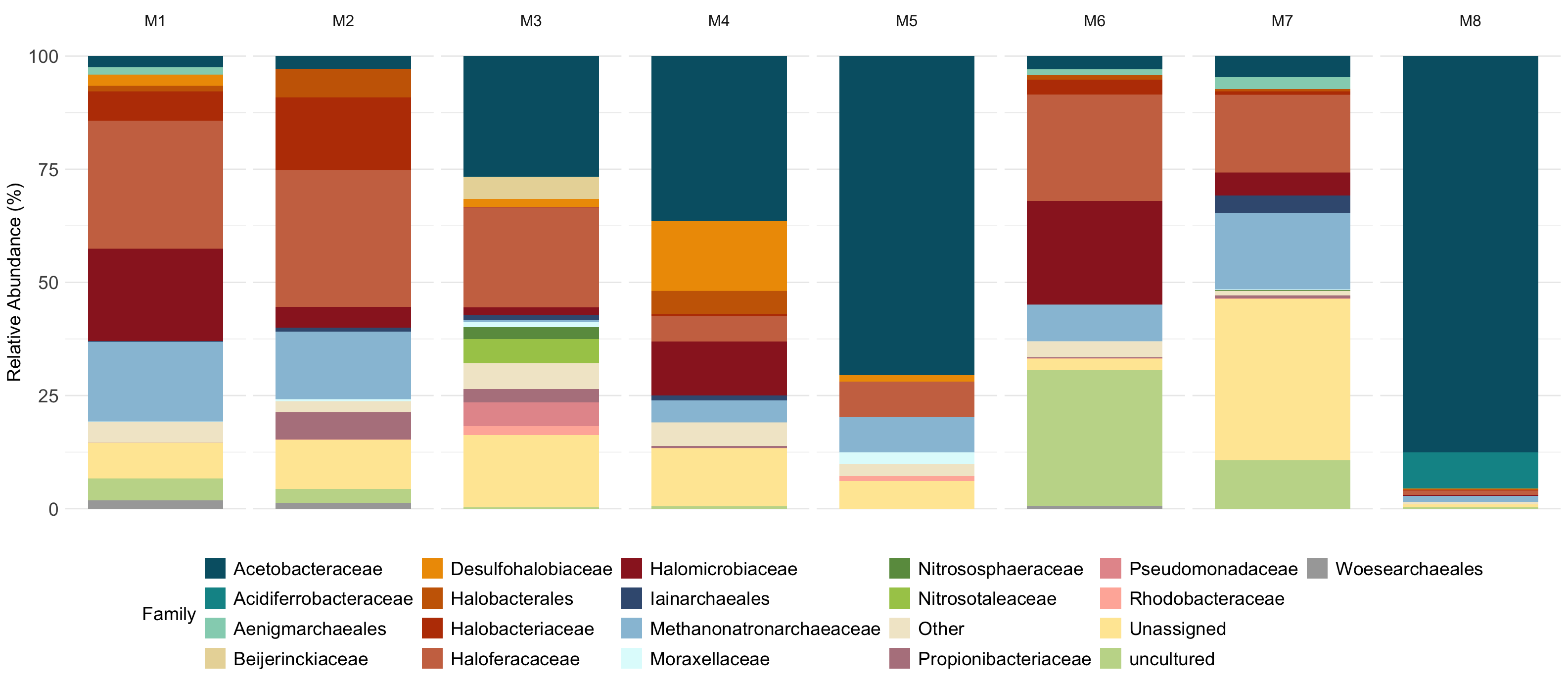

Supplement: Supplementary Figure S1 — Family-level taxonomy. Relative abundance of 20 most abundant families across samples. [file Image_1.TIFF]
